# Investigating the Secondary Care System Burden of Glycogen Storage Disease Type Ia (GSDIa) Using the Hospital Episode Statistics Database

**DOI:** 10.36469/001c.137126

**Published:** 2025-05-28

**Authors:** Eliza Kruger, Shreena Giblin

**Affiliations:** 1 Ultragenyx Pharmaceutical, Inc., Novato, California, USA; 2 IQVIA, London, UK

**Keywords:** glycogen storage disease, quality of life, burden of disease

## Abstract

**Background:** Glycogen storage disease type Ia (GSDIa) is a rare, inherited metabolic disorder characterized by a deficiency in glucose 6-phosphatase. People living with GSDIa are at high risk for clinical manifestations (including hypoglycemia and hepatomegaly) and clinical complications (including hyperlipidemia, stunted growth, liver adenomas, and renal failure). Evaluating symptom management and secondary care burdens is vital to understanding the patient experience and optimizing care pathways. **Objective:** We sought to quantify the number of patients with GSDIa within secondary care settings across England and to evaluate the burden of disease associated with living with GSDIa. **Methods:** This study utilized the United Kingdom Hospital Episode Statistics (HES) database across a 69-month time period (April 2015–December 2020) to investigate National Health Service (NHS) resource use and GSDIa mortality. **Results:** Patients (N = 943) with GSDIa were identified. Frequent manifestations included anemia (n = 421; 45%), hypoglycemia (n = 185; 20%), and hepatomegaly (n = 152; 16%). On average, patients had a total of 8 events/year, including 2 elective events, 2 nonelective emergencies, 1 outpatient visit, and 3 daycase visits. In the entire HES population, there was approximately 1 (~60% elective, ~40% nonelective) event/year. The highest total number of events across the entire patient journey tracked within the HES occurred with adolescents (12-17 years) who had an average of 28.5 events. Average length of stay was greatest in the pediatric infantile (0-2 years) population with 4.6 days and 3.4 days for nonelective and elective events, respectively. When benchmarked against the general population, patients with GSDIa had a mortality rate of 4.3%, compared with 0.9% for the entire HES population. The average age at mortality was 14.3 years lower for patients with GSDIa vs the entire HES population (63.7 years vs 78.0 years). **Discussion:** This study demonstrates high burden associated with GSDIa. Complications are a key driver of NHS resource use. Mortality associated with GSDIa in hospitalized patients is higher than the general population. **Conclusions:** GSDIa imposes a large burden on the healthcare system. There is a clear unmet need for patients with GSDIa, and complications are a substantial driver of resource use and burden of disease.

Glycogen storage diseases (GSDs) are a group of rare genetic disorders characterized by an enzymatic defect that results in the failure to synthesize or metabolize glycogen.^1,2^ Glycogen storage disease type Ia (GSDIa) is caused by a deficiency of glucose-6-phosphatase (G6Pase-α), a key enzyme that catalyzes the final step in glycogenolysis and gluconeogenesis.^3,4^ The conversion of glucose-6-phosphate (G6P) to free glucose, catalyzed by G6Pase-α, is a key step in releasing glucose from the liver into the bloodstream; the absence of G6Pase-α causes patients with GSDIa to suffer from serious and potentially life-threatening episodes of hypoglycemia.^5,6^

G6P is also a metabolite involved in multiple metabolic pathways, and accumulation of G6P can lead to other imbalances and/or excessive accumulation of other metabolites, such as lactic acid, uric acid, lipids, and triglycerides.^7^ Glycogen accumulation in both the liver and kidneys can also lead to hepatomegaly and nephromegaly, both hallmarks of GSDIa.^6^

There are no approved treatments for GSDIa, and the current standard of care involves strict dietary management with either frequent feedings of uncooked or modified cornstarch or gastric drip feeding of glucose through the night, a method frequently used for pediatric patients.^8–10^ Due to the rarity of the condition, there are few specialty centers of care, and the absence of specific guidelines leads to heterogeneity in care across secondary-care providers.

It was hypothesized that individuals living with GSDIa have a significant number of complications and require substantially more secondary care than individuals without GSDIa. The aim of this study was to quantify the number of patients with GSDIa within secondary care across England and to evaluate the burden of disease, including morbidity and mortality, associated with living with GSDIa. The primary objectives of this study were to characterize the patient journey of GSDIa in secondary care, to evaluate resource use and disease burden for specific patient subgroups to better understand the secondary care experience of patients with GSDIa, and to understand mortality associated with GSDIa in England.

## METHODS

### Study Design and Data Sources

This was a retrospective cohort study with all analyses conducted using the UK Hospital Episode Statistics (HES) database.^11^ This database contains *International Classification of Disease, Tenth Revision* (ICD-10)–coded records for the primary and secondary diagnostic reasons for admissions to all National Health Service (NHS) hospitals in England; these records are routinely collected within the NHS for administrative purposes. HES captures activity across inpatient, outpatient, and accident/emergency settings. Patient details such as age, comorbidities, and any procedures they have undergone are recorded.

HES data are maintained in the IQVIA – Woking, United Kingdom office according to strict, best-practice information governance protocols in agreement with NHS Digital.^11^ HES data are a nationally collected data set representative of the entire English population, and HES data cleaning rules can be found on the NHS Digital website under HES.^11^

### Cohort Construction

HES data from 69 months (April 2015–December 2020) were used to extract all inpatient and outpatient episodes for patients who had a diagnosis of GSD and met the inclusion criteria. The ICD-10 code E740 was used to initially identify patients with all GSD subtypes. Due to the current coding structure used by the NHS, this code was only available at the 4-character level and was therefore insufficient to specifically identify patients with GSDIa. To add further sensitivity specific to our patient selection process, we conducted a review by 2 experts with experience in GSD, who evaluated ICD-10 codes commonly encountered in GSD patients. These experts determined which codes were indicative of GSDIa, as opposed to other GSD subtypes such as GSDIb or GSDIII, ensuring a more accurate identification of patients with GSDIa. This expert review process was designed to mitigate the limitations of the 4-character ICD-10 code and enhance the specificity of our cohort selection. In addition, ICD-10 codes related to complications associated with GSDIa were also used as patient inclusion criteria for this study. These complications were identified through literature searches and input from internal experts. The identification of the inclusion cohort was performed in parallel with identification of an exclusion cohort based on complications associated with other GSD subtypes. Both inclusion and exclusion codes are listed in **Supplementary Table S1**. The overall HES population was used as a comparator group.

### Event Definitions

Events associated with care were categorized as follows:

- **Elective events:** Planned hospital admissions where patients were scheduled in advance, usually for non-emergency treatments or procedures
- **Nonelective events:** Unplanned hospital admissions that occurred due to urgent or emergency medical conditions requiring immediate care
- **Daycase:** A hospital admission where the patient underwent a planned procedure or treatment and was discharged on the same day without an overnight stay
- **Outpatient:** Medical care or treatment provided in a hospital or clinic where the patient is not admitted and is returned home after the appointment

### Statistical Methods

Descriptive analysis was performed using number and percent within each category; total patient and event counts, mean (with 95% confidence intervals) for continuous variables, and categorical variables were graphically presented as bar charts and tables. All values for comorbid groups (ie, identified GSDIa patients with a specific comorbidity also recorded) were compared against the non-comorbid cohort (ie, identified GSDIa patients without the specified comorbidity).

## RESULTS

A total of 943 patients with GSDIa met the relevant criteria for inclusion and had data recorded within the HES across the time window from April 2015 to December 2020. The mean age of the GSDIa cohort was 35.5 years; 59% were male. The mean (SD) age and proportion of males in each age band were as follows: 0-2 (n = 165), 0.5 (0.7) years, 67% male; 3-11 (n = 175), 6.3 (2.5) years, 64% male; 12-17 (n=64), 14.5 (1.6) years, 55% male; 18+ (n = 539), 48.3 (18.5) years, 56% male.

### Inpatient and Outpatient Events

Patients entered the study at different time points, resulting in varied study periods. Patients were recorded with a total of 22 667 events during the analytical time window. Outpatient events accounted for 40% of total events (n = 9164), nonelective events for 30% (n = 6705), elective events for 19% (n = 4407), and daycases for 11% (n = 2391). Adult patients had the highest proportion of elective and nonelective events associated with their care (27% [n = 3319] and 35% [n = 4206], respectively) while patients in the pediatric and adolescent age bands had the highest proportion of outpatient events associated with their care (56% [n = 2412] and 57% [n = 2483] and 51% [n = 931], respectively). Patients in the 12-17 age band had the highest proportion of daycases associated with their care (16% [n = 295] (**Table 1**).

**Table 1.** Total Events By Care Setting

**Care Setting**	**Age Band (y)**				
	**0-2 (n = 165)**	**3-11 (n = 175)**	**12-17 (n = 64)**	**18+ (n = 539)**	**All (n = 943)**
Elective	411 (10)	532 (12)	145 (8)	3319 (27)	4407 (19)
Nonelective	1292 (30)	752 (17)	455 (25)	4206 (35)	6705 (30)
Daycase	233 (5)	568 (13)	295 (16)	1295 (11)	2391 (11)
Outpatient	2412 (56)	2483 (57)	931 (51)	3338 (28)	9164 (40)

On average, patients had 8 events per year, composed of 2 elective events, 2 nonelective events, 1 daycase, and 3 outpatient events. Patients in the pediatric infantile age band (0-2 years) had the greatest average number of events associated with their care per year (9 events in total) (**Table 2**).

**Table 2.** Average Events per Year by Care Setting

	**Age Band (y)**				
	**0-2 (n = 165)**	**3-11 (n = 175)**	**12-17 (n = 64)**	**18+ (n = 539)**	**All (n = 943)**
Average events per patient					
Elective	2.5	3.0	2.3	6.2	4.7
Nonelective	7.8	4.3	7.1	7.8	7.1
Daycase	1.4	3.2	4.6	2.4	2.5
Outpatient	14.6	14.2	14.5	6.2	9.7
Total	26.3	24.7	28.5	22.6	24.0
Average events per patient per year					
Elective	0.8	0.9	0.6	2.3	1.6
Nonelective	2.6	1.3	1.9	2.9	2.4
Daycase	0.5	1.0	1.2	0.9	0.9
Outpatient	4.9	4.3	3.9	2.3	3.3
Total	8.8	7.5	7.6	8.4	8.2

**Figure 1.**
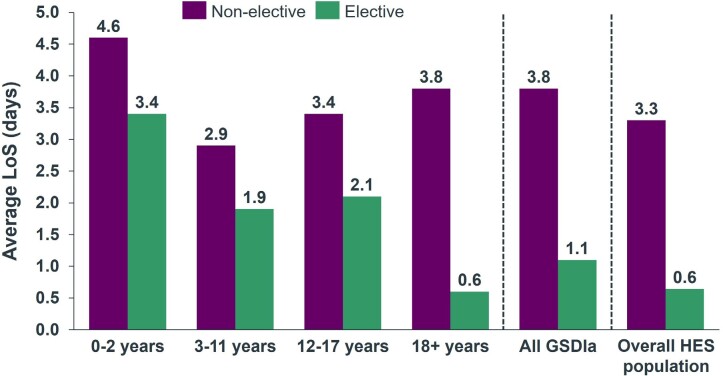
Average Length of Stay per Patient per Event Abbreviations: GSDIa, glycogen storage disease type Ia; HES, Hospital Episode Statistics; LoS, length of stay.

Across the analytical time window, the average length of stay was 1.1 days for elective events and 3.8 days for nonelective events. The highest average length of stay was associated with the 0-2 pediatric age band for both nonelective (4.6 days) and elective care (3.4 days) (**Figure 1**).

### Complications

**Figure 2.**
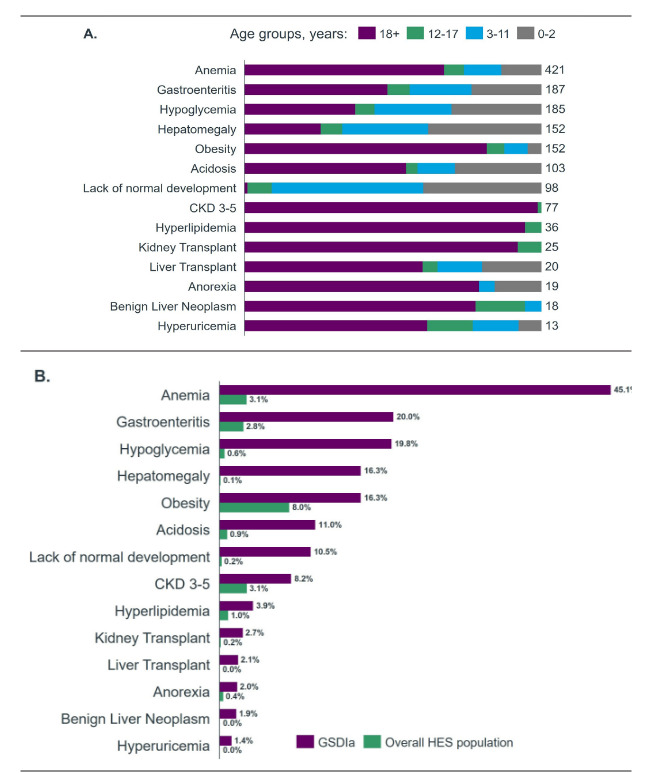
Evaluation of GSDIa-Related Complications Complication patient counts by age band in the GSDIa cohort.Frequency of complications in the GSDIa cohort compared with the overall HES population. Abbreviations: CKD, chronic kidney disease; GSDIa, glycogen storage disease type Ia; HES, Hospital Episode Statistics.

Across the analytical time window, patients were tracked for a total of 14 distinct complications. The most common complications recorded within the GSDIa cohort were anemia (n = 421), gastroenteritis (n = 187), and hypoglycemia (n = 185). The least common complications included hyperuricemia (n = 13), benign liver neoplasms (n = 18), and anorexia (n = 19) (**Figure 2A**). Patients with GSDIa had a higher incidence of all complications considered compared with the overall HES population (**Figure 2B**). Patients with GSDIa had an average of 3 secondary care events per patient per year with recorded complications. For the total time a patient was tracked in the study, the average number of events per patient was 8.7 events (**Table 3**).

**Table 3.** Average Events Including a Recorded Complication per Year

	**Age Band (y)**				
	**0-2 (n = 165)**	**3-11 (n = 175)**	**12-17 (n = 64)**	**18+ (n = 539)**	**All (n = 943)**
Total complication events	870	1057	462	5847	8236
Events per patient (average)	5.3	6.0	7.2	10.8	8.7
Events per year (average)	1.9	1.9	2.2	4.3	3.0

### Mortality

The mortality rate for patients with GSDIa was 3.4% higher than the recorded mortality for the wider population recorded in HES (not just patients with GSDIa): 4.3% vs 0.93%, respectively. In addition, the average age at mortality for patients with GSDIa was 14.3 years lower than that of the overall HES population (63.7 years vs 78.0 years (**Table 4**).

**Table 4.** Stratified Mortality Captured in the HES Database in England by Gender and Age Band

	**Age Band (y)**				
	**0-2**	**3-11**	**12-17**	**18+**	**All**
Mortality rate: GSDIa (%)	1.87	1.64	0	6.74	4.3
No.	2	3	0	39	44
Average age at mortality (y)	0	6.7	–	67.7	63.7
Mortality rate: Overall HES population (%) (2015-2020)	0.14	0.01	0.01	1.17	0.93
No.	17 199	2 949	3 239	3 244 921	3 268 308
Average age at mortality (y)	0.12	4.5	15.1	78.6	78.0

Abbreviations: GSDIa, glycogen storage disease type Ia; HES, Hospital Episode Statistics.

## DISCUSSION

There is a high burden associated with GSDIa. Patients had several different complications associated with their condition and a high degree of resource use within the analytical time window measured. Compared with the general population across the entire HES data set, patients with GSDIa had a significantly greater number of events associated with their care. Overall, patients with GSDIa had an average of 4.9 inpatient events and 3.3 outpatient events per year, compared with patients without GSDIa with 1.0 inpatient event and 1.7 outpatient events per year. Of the average of 8.2 total events per year in patients with GSDIa, 3 were related to GSDIa-specific complications.

The most frequent complications associated with GSDIa in our retrospective study (ie, anemia, gastroenteritis, and hypoglycemia) are consistent with the known clinical manifestations of the disease.^12,13^ We found that children and adolescents with GSDIa more commonly experienced acute complications of the disease (eg, hypoglycemia and poor growth), while adults more commonly experienced long-term complications (eg, chronic kidney disease). These results are consistent with findings from a retrospective analysis of the PharMetrics® Plus claims database, which assessed complications in 557 patients with GSDIa vs 5570 matched non-GSDIa comparators in the United States.^14^ Our study also found that patients with GSDIa had high healthcare resource utilization, including longer average length of stay per hospital admission vs comparators, which is consistent with findings from the PharMetrics claims database study,14 as well as with findings from a separate retrospective study evaluating healthcare resource utilization among 1402 patients with GSDIa and 1402 matched non-GSDIa comparators in the US using the PearlDiverTM claims database with the Mariner data set.^15^

Our research also confirms that, despite current management with cornstarch, patients with GSDIa are at an increased risk of mortality compared with the general population. Our findings are also consistent with the work by Derks et al that showed a significant unmet need for new treatments to better manage disease symptoms in patients with GSDIa.^13^

There is also a dearth of literature on mortality in patients with GSDIa. Contrary to other evidence (ie, the report by the National Organization for Rare Disorders) that indicates early diagnosis and treatment can result in normal growth and puberty, with many affected individuals living into adulthood and enjoying normal life activities,^16^ we found significantly higher mortality in patients with GSDIa compared with the general population.

This study focused on burden of disease in terms of healthcare resource utilization. Other studies have shown significant quality-of-life impacts and high burden of disease from the patient perspective.^17–19^

### Limitations

A key limitation of this study is a lack of an ICD-10 code for GSDIa that may have resulted in inclusion of other GSDs. Equally, there may have been instances where patients were not recorded with the ICD-10 code. Alternatively, patients could have been recorded with the ICD-10 code, but without a record of associated complications. In both cases, these patients would have been excluded from all or certain parts of the analyses; however, this is likely to be a low number of patients. Outpatient appointments are also often not given diagnostic codes because they are not required for reimbursement, which may result in underestimation of the impact of these types of visits. Since primary care plays a critical role in disease management, future studies incorporating general practice and prescription data would provide a more holistic view of disease impact.

There can also be an overlap in complications for different types of GSDs (eg, GSDIII and GSDIX), such as hypoglycemia and hepatomegaly. This would have resulted in other types of GSDs being included in our study. However, this study took a conservative approach to identifying patients with multiple inclusion and exclusion criteria; therefore, this number is also likely to be small. Future studies incorporating genetic or biomarker-confirmed diagnosis could help further validate findings. The specified time window in the HES database may not have accurately informed complete patient history, and we may have missed certain activity and complications due to the longitudinal nature of the data set.

GSDIa is a lifelong, chronic condition; however, we only analyzed 5 years and 9 months of data, which may not fully capture the long-term burden of disease. A longer analytical time window would have enabled a more robust evaluation of disease burden, particularly for older patients who are likely to have had multiple care touchpoints in secondary care prior to the evaluated time window. Finally, we were unable to analyze burden in primary care for this group of patients, which could have provided a more complete picture of overall disease burden, because these data were not recorded within the HES data set.

### Implications

The higher mortality rate and younger age at death observed in patients with GSDIa underscore the need for improved disease management strategies. While early diagnosis and optimized dietary interventions can help mitigate some risks, our findings highlight an unmet need for more effective treatment options that address the underlying cause of disease. Additionally, standardizing care pathways and increasing access to specialized metabolic centers could potentially reduce disease-associated morbidity and mortality.

Key recommendations include enhancing diagnostic coding to better capture rare diseases in hospital databases, developing standardized treatment guidelines to ensure consistent care across centers, and expanding access to metabolic specialists. Additionally, novel therapeutic approaches, such as gene therapy, could transform the management of GSDIa and reduce long-term complications.^20,21^ Policymakers and healthcare providers should prioritize these strategies to improve patient care and reduce the healthcare burden associated with this rare disease.

## CONCLUSIONS

GSDIa imposes a large burden on the healthcare system. The pediatric infantile age band (0-2 years) had the greatest average number of events associated with their care per year and the longest average hospital length of stay. The mortality rate was approximately 3.4% higher in the overall GSDIa cohort vs the overall HES population, and the average age at mortality was more than 14 years lower in the GSDIa group. Increased mortality in patients with GSDIa was also observed in the 0-2, 3-11, and 18+ age bands compared with the national benchmark for these ages. There is a clear unmet need for patients with GSDIa, and complications are a substantial driver of resource use and burden of disease.

### Competing Interests

E.K. is currently employed by Moderna. The work presented here was conducted while employed at Ultragenyx and was not influenced by the author’s current employer. S.C. declares no competing interests.

### Ethics Approval and Consent to Participate

Hospital Episode Statistics is a retrospective patient-anonymized data set. Ethics approval is not required.

### Availability of Data and Materials

The data sets used and analyzed in this study are available from the corresponding author on reasonable request.

## Supplementary Material

Online Supplementary Material

## References

[ref-449856] Burda P., Hochuli M. (2015). Hepatic glycogen storage disorders: what have we learned in recent years?. Curr Opin Clin Nutr Metab Care.

[ref-449857] Shin Y. S. (2006). Glycogen storage disease: clinical, biochemical, and molecular heterogeneity. Semin Pediatr Neurol.

[ref-449858] Lei K. J., Shelly L. L., Lin B.. (1995). Mutations in the glucose-6-phosphatase gene are associated with glycogen storage disease types 1a and 1aSP but not 1b and 1c. J Clin Invest.

[ref-449859] Lei K. J., Shelly L. L., Pan C. J., Sidbury J. B., Chou J. Y. (1993). Mutations in the glucose-6-phosphatase gene that cause glycogen storage disease type 1a. Science.

[ref-449860] Bruni N., Rajas F., Montano S., Chevalier-Porst F., Maire I., Mithieux G. (1999). Enzymatic characterization of four new mutations in the glucose-6 phosphatase (G6PC) gene which cause glycogen storage disease type 1a. Ann Hum Genet.

[ref-449861] Chou J. Y., Jun H. S., Mansfield B. C. (2015). Type I glycogen storage diseases: disorders of the glucose-6-phosphatase/glucose-6-phosphate transporter complexes. J Inherit Metab Dis.

[ref-449862] Rajas F., Gautier-Stein A., Mithieux G. (2019). Glucose-6 phosphate, a central hub for liver carbohydrate metabolism. Metabolites.

[ref-449863] Shah K. K., O’Dell S. D. (2013). Effect of dietary interventions in the maintenance of normoglycaemia in glycogen storage disease type 1a: a systematic review and meta-analysis. J Hum Nutr Diet.

[ref-449864] Correia C. E., Bhattacharya K., Lee P. J.. (2008). Use of modified cornstarch therapy to extend fasting in glycogen storage disease types Ia and Ib. Am J Clin Nutr.

[ref-449865] Greene H. L., Slonim A. E., O’Neill J. A., Jr., Burr I. M. (1976). Continuous nocturnal intragastric feeding for management of type 1 glycogen-storage disease. N Engl J Med.

[ref-449866] NHS Digital Hospital Episode Statistics (HES).

[ref-449867] Bali D. S., El-Gharbawy A., Austin S.., Adam M. P., Feldman J., Mirzaa G. M.. (1993). GeneReviews®.

[ref-449868] Derks T. G. J., Rodriguez-Buritica D. F., Ahmad A.. (2021). Glycogen storage disease type Ia: current management options, burden and unmet needs. Nutrients.

[ref-449869] Kruger E., Nedzesky J., Thomas N., Dunn J. D., Grimm A. A. (2025). Glycogen storage disease type Ia: a retrospective claims analysis of complications, resource utilization, and cost of care. J Health Econ Outcomes Res.

[ref-449870] Nedzesky J., Kruger E., Gupta R. N., Thomas N. A., Valayannopoulos V. (2022). Healthcare resource utilization among patients with glycogen storage disease type Ia (GSDIa) in the United States and the impact of COVID-19: a claims database analysis. Value Health.

[ref-449871] National Organization for Rare Disorders (NORD) Glycogen storage disease type I.

[ref-449872] Kruger E., Aggio D., de Freitas H., Lloyd A. (2023). Estimation of health utility scores for glycogen storage disease type Ia. Pharmacoeconomics Open.

[ref-449873] Butler J., Dress A., Theodore-Oklota C.. (2020). The humanistic burden of glycogen storage disease type Ia: the impact on symptoms, diet and health-related quality of life. Value Health.

[ref-449874] Kruger E., de Freitas H. M., Ferrecchia I., Graydon M., Lloyd A. (2025). People and families affected by glycogen storage disease type Ia: an analysis of narrative accounts written by individuals living with GSDIa and their caregivers. J Health Econ Outcomes Res.

[ref-449875] Chou J. Y., Mansfield B. C. (2023). Gene therapy and genome editing for type I glycogen storage diseases. Front Mol Med.

[ref-449876] Weinstein D. A., Derks T. G., Rodriguez-Buritica D. F.. (2025). Safety and efficacy of DTX401, an AAV8-mediated liver-directed gene therapy, in adults with glycogen storage disease type I a (GSDIa). J Inherit Metab Dis.

