# Supplementary material for: Investigating the Secondary Care System Burden of Glycogen Storage Disease Type Ia (GSDIa) Using the Hospital Episode Statistics Database
Source: J Health Econ Outcomes Res. 2025 May 28;12(1):201–6. doi: 10.36469/001c.137126 (PMC12124281; doi:10.36469/001c.137126)
Supplement: Online Supplementary Material [file jheor_2025_12_1_137126_285018.pdf]

Journal of Health Economics and Outcomes Research

Online Supplementary Material

Investigating the Secondary Care System Burden of Glycogen Storage Disease Type Ia Using the Hospital Episode Statistics Database. *JHEOR*. 2025;12(1):201-206. doi:10.36469/jheor.2025.137126

Table S1: Inclusion/Exclusion Codes for Patients

This supplementary material has been provided by the authors to give readers additional information about their work.

This is an open-access article distributed under the terms of the Creative Commons Attribution 4.0 International License (CCBY-4.0). View this license's legal deed at http://creativecommons.org/licenses/by/4.0 and legal code at http://creativecommons.org/licenses/by/4.0/legalcode for more information.

Table S1. Inclusion/Exclusion Codes for Patients

Complication/ Comorbidity Type	Code	Type	Inclusion/ Exclusion	Notes	References
Anemia	D550, D509, D638, D508	ICD-10	Inclusion	Indicative of GSDIa	Wang et al (2012)
Acidosis	P740, E872	ICD-10	Inclusion	Indicative of GSDIa	Bali et al (1993)<br>Kishnani et al (2014)<br>Nirzar et al (2021)
Benign liver neoplasm	D134	ICD-10	Inclusion	Indicative of GSDIa	Bali et al (1993)<br>Baheti et al (2015)
CKD 3-5	N183, N184, N185	ICD-10	Inclusion	Indicative of GSDIa	Bali et al (1993)<br>Chen et al (1991)<br>Martens et al (2009)
Gastroenteritis	A099	ICD-10	Inclusion		Bali et al (1993)
Hepatomegaly	R160, R162, K778	ICD-10	Inclusion	Indicative of GSDI	Kishnani et al (2014)
Hyperlipidemia	E782, E785	ICD-10	Inclusion	Indicative of GSDIa	Bali et al (1993)<br>Carvalho et al (2013
Hyperuricemia	E790	ICD-10	Inclusion	Indicative of GSDIa	Kishnani et al (2014)
Hypoglycemia	E162	ICD-10	Inclusion	Indicative of GSDI	Kishnani et al (2014)<br>Nirzar et al (2021)
Lack of normal development	R620, R628, R629	ICD-10	Inclusion	Indicative of GSDI	Nirzar et al (2021)
Obesity	E668, E669	ICD-10	Inclusion	Indicative of GSDIa	Melis et al (2015)
Osteoporosis	M819	ICD-10	Inclusion	Indicative of GSDIa	Cabrera–Abreu et al (2004)
Dietary counseling and management	Z713	ICD-10	Inclusion	Indicative of GSDI	–
Personal history of endocrine, nutritional, and metabolic disorders	Z863	ICD-10	Inclusion	Indicative of GSDI	–
Dialysis	X40	OPCS	Inclusion	Indicative of GSDIa	Bali et al (1993)
IBD	K58	ICD-10	Exclusion	Indicative of GSDIb	Lawrence et al (2015)
Cardiomyopathy	I42, I43	ICD-10	Exclusion		Austin et al (2013)
Other specified congenital malformations of heart	Q24.8	ICD-10	Exclusion	Indicative of GSDIII	Austin et al (2013)
Other specified disorders of muscle	M62.8	ICD-10	Exclusion	Indicative of GSDIII	Martin et al (1993), Preisler et al (2013), Herbert et al (2018)
Muscle strain (fatigue)	M62.6	ICD-10	Exclusion	Indicative of GSDIII, GSDV, GSDIX	Martin et al (1993), Preisler et al (2013), Herbert et al (2018)
Fibrosis and cirrhosis of liver	K74	ICD-10	Exclusion	Indicative of GSDIV	Magoulas et al (2013)
Metabolic disorder drugs	X91	OPCS	Exclusion	Indicative of Pompe disease	–

Abbreviations: CKD, chronic kidney disease; GSD, glycogen storage disease; IBD, irritable bowel disease; ICD-10, *International Classification of Diseases, Tenth Revision*; OPCS, Office of Population Censuses and Surveys Classification of Surgical Operations and Procedures.
